# Supplementary material for: Bedsharing among breastfeeding physicians: Results of a nationwide survey
Source: PLoS One. 2024 Aug 1;19(8):e0305625. doi: 10.1371/journal.pone.0305625 (PMC11293648; doi:10.1371/journal.pone.0305625)
Supplement: S1 Appendix — (DOCX) [file pone.0305625.s002.docx]

S1 Appendix. Physician mothers/birthing people infant feeding and sleep survey

Start of Block: TELL US A LITTLE BIT ABOUT YOU

Q46 Thank you for taking the time to help us learn more about the experience physicians have with infant feeding and sleep. Physicians face unique challenges with infant feeding and sleep. Through your participation in this survey, we hope to learn more about how to optimize the early parenting experiences for physicians during the most strenuous time in training or practice. **If you have birthed multiple children, please answer all of the questions for the child you had during the most strenuous time of your training or practice**

Q1 Physician or Physician in training

- Yes (1)
- No (2)

Skip To: End of Survey If Physician or Physician in training != Yes

Q2 Singleton pregnancy

- Yes (1)
- No (2)

Skip To: End of Survey If Singleton pregnancy    != Yes

Q48 **If you have multiple children, please answer all of the questions for the child you had during the most strenuous time of your training or practice.**

Q4 Identify the most strenuous time of your medical career during which you had a child

▼ Medical school (1) ... Other (7)

Q5
If you answered other, in the above question, please tell us about it.

________________________________________________________________

Q6 Specialty

▼ Allergy and Immunology (1) ... Other (21)

Q41 If you responded "Other" in the previous question, please write your Specialty.

________________________________________________________________

Q7 Race

▼ White (1) ... Other, please write down (7)

Q8 If you answered other, for the above question, please tell us about it

________________________________________________________________

Q9 Spanish/Hispanic Ethnicity

▼ No, Not Spanish/Hispanic (1) ... Other Spanish or Hispanic (6)

| 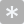 |
| --- |

Q10 Number of Children

________________________________________________________________

| 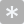 |
| --- |

Q11 Medical school graduation year

________________________________________________________________

Q12 In which state do you currently reside

▼ Alabama (1) ... I do not reside in the United States (53)

Q13 Relationship status while breastfeeding

▼ Married (1) ... Domestic Partnership (7)

End of Block: TELL US A LITTLE BIT ABOUT YOU

Start of Block: YOUR BABY'S BIRTH AND FEEDING

Q45 BIRTH AND FEEDING QUESTIONS

Q43 What year was your baby born?

________________________________________________________________

Q14 How was your baby delivered

▼ Vaginally and not induced (1) ... An unplanned or emergency cesarean (4)

| 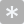 |
| --- |

Q15  At what gestational age (weeks) was your baby delivered

________________________________________________________________

Q16 Did you initiate breastfeeding

- Yes (1)
- No (2)

Skip To: Q22 If Did you initiate breastfeeding = No

Q17 About how long after your delivery did you breastfeed or try to breastfeed your baby for the very first time

▼ Less than 30 minutes (1) ... More than 2 days (9)

Q18  How old (in months) was your baby when you completely stopped breastfeeding and expressing milk. Respond in box still breastfeeding if applicable.

________________________________________________________________

Q19 After your milk came in, how long did you exclusively breastfeed? (Disregard any medically indicated supplementation while establishing initial milk)

▼ Less than 1 month (1) ... Greater than 8 months (10)

Q20 Did you breastfeed as long as you wanted to?

- Yes (1)
- No (2)

Q21 How important was each of the following reasons for your decision to feed your baby formula or stop breastfeeding? Skip if still breastfeeding.

|  | Not important at all (1) | Not very important (2) | Somewhat important (3) | Very important (4) |
| --- | --- | --- | --- | --- |
| I desired weaning (1) |  |  |  |  |
| My baby had trouble sucking or latching on (3) |  |  |  |  |
| My baby became sick and could not breastfeed (4) |  |  |  |  |
| My baby began to bite (5) |  |  |  |  |
| My baby lost interest and began to wean him or herself (6) |  |  |  |  |
| My baby was old enough that the difference between breast milk an formula no longer mattered (7) |  |  |  |  |
| Breast milk did not satisfy my baby (8) |  |  |  |  |
| Breastfeeding was too painful (9) |  |  |  |  |
| Breastfeeding was too inconvenient (10) |  |  |  |  |
| Breastfeeding was too tiring (11) |  |  |  |  |
| My nipples were sore, cracked or bleeding (12) |  |  |  |  |
| My breasts were overfull or engorged (13) |  |  |  |  |
| My breasts were infected or abscessed (14) |  |  |  |  |
| My breasts leaked too much (15) |  |  |  |  |
| I did not like breastfeeding (16) |  |  |  |  |
| I was sick or had to take medicine (17) |  |  |  |  |
| I wanted to be able to leave my baby for several hours (18) |  |  |  |  |
| I wanted to go back to my usual diet (19) |  |  |  |  |
| I wanted to smoke again or more than I did while breastfeeding (20) |  |  |  |  |
| I had too many household duties (21) |  |  |  |  |
| I could not or did not want to pump or breastfeed at work (22) |  |  |  |  |
| Pumping milk no longer seemed worth the effort that it required (23) |  |  |  |  |
| I was not present to feed my baby for reasons other than work (24) |  |  |  |  |
| Someone else wanted to feed my baby (25) |  |  |  |  |
| I did not want to breastfeed in public (26) |  |  |  |  |
| I wanted by body back to myself (27) |  |  |  |  |
| I became pregnant or wanted to become pregnant again (28) |  |  |  |  |
| Other (29) |  |  |  |  |

Q22   Did you express milk for this baby

- Yes (1)
- No (2)

Skip To: Q26 If   Did you express milk for this baby = No

Q23 For what reasons did you express milk for this baby, select all that apply

- To increase my milk supply (1)
- Because I went back to work (2)
- Because I was traveling without my baby (3)
- Because I was engorged / had too much milk (4)
- So that someone else could feed my baby (5)
- To store extra breast milk (6)
- Because my baby had trouble latching (7)
- Because my nipples were sore (8)
- Because I prefer to feed my baby breast milk from a bottle (9)
- Other (10) ________________________________________________

Q24 If you expressed breastmilk at work, where was the primary location of milk expression?

- Private call room (1)
- Designated lactation room (2)
- Shared workspace ( e.g staff lounge, physician workroom) (3)
- Other, please explain (4) ________________________________________________

Q25 At what age did your baby stop latching. Respond in box still breastfeeding if applicable

________________________________________________________________

| 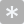 |
| --- |

Q26 How long was your maternity leave ( in weeks)

________________________________________________________________

| Page Break |  |
| --- | --- |

End of Block: YOUR BABY'S BIRTH AND FEEDING

Start of Block: YOUR BABY’S SLEEPING HABITS

Q46 SLEEP QUESTIONS

Q27 Where did your baby usually sleep at night

|  | 0-6 months (1) | 7-12 months (2) |
| --- | --- | --- |
| In your room (1) |  |  |
| In a different room (2) |  |  |

Q28 What did your baby usually sleep in if they slept in the same room as you, select one answer for each time period. **Skip if your baby did not sleep in the same room as you**

|  | 0-3 months (1) | 4-6 months (2) | 7-12 months (3) |
| --- | --- | --- | --- |
| Crib (1) |  |  |  |
| In bed with you (2) |  |  |  |
| On a couch, sofa, or armchair (3) |  |  |  |
| In a swing (4) |  |  |  |
| Cosleeper (attaches to side of your bed) (5) |  |  |  |
| Bassinet (6) |  |  |  |
| Car seat (7) |  |  |  |

Q29 On the night that you laid down with or slept with your baby, did you usually have the baby with you all night or part of the night. **Skip if your baby did not sleep in the same room as you**

|  | 0-3 months (1) | 4-6 months (2) | 7-12 months (3) |
| --- | --- | --- | --- |
| All night (1) |  |  |  |
| The first part of the night only (2) |  |  |  |
| The last part of the night only (3) |  |  |  |
| Several short times throughout the night (4) |  |  |  |

Q30 How many nights per week did you and your baby usually lie down together or sleep together. **Check never if baby did not sleep in the same room as you**

|  | 0-3 months (1) | 4-6 months (2) | 7-12 months (3) |
| --- | --- | --- | --- |
| Always (1) |  |  |  |
| Sometimes (2) |  |  |  |
| Never (3) |  |  |  |

Q31 When you and your baby laid down together or slept together, did you usually: ( Skip if your baby did not sleep in the same room as you)

|  | 0-3 months (1) | 4-6 months (2) | 7-12 months (3) |
| --- | --- | --- | --- |
| Stay with the baby and also sleep (1) |  |  |  |
| Keep awake until the baby was asleep or finish feeding, and then put the baby somewhere else while you slept? (2) |  |  |  |

Q32 On the night that you and your baby laid down together or slept together, who else usually laid down with or slept with you ? (Please check all that apply).  Skip if your baby did not sleep in the same room as you

- Your husband or partner (1)
- Your other child or children (2)
- Other people (3)
- No one else (4)

Q33 What were your reasons for bringing your baby to bed with you (Please check all that apply)  Skip if your baby did not sleep in the same room as you

- Not applicable, I did not bring my baby to bed with me (11)
- It was commonly done in my family (1)
- Sleeping with my baby helped the baby or me to sleep better (2)
- I though it was safer if my baby slept with me or us (3)
- A doctor or nurse advised sleeping with my baby to breastfeed (4)
- To breastfeed (5)
- To bottle feed (6)
- To help with a blocked milk duct or other breastfeeding problem (7)
- To be close or bond (8)
- To comfort when fussy (9)
- To comfort when sick (10)
- Other (12)

Q34 What were your reasons for not bringing your baby to bed with you (Please check all that apply)

- Not applicable, I brought my baby to bed with me (7)
- It was not commonly done in my family (1)
- We woke each other up, or baby woke me or others in the bed (2)
- I thought it was safer if my baby did not sleep with me or us (3)
- I did not think the baby should sleep with me because I smoke, take sedative medicine or other reason (4)
- A doctor or nurse advised not sleeping with my baby (5)
- I thought it would be too hard to get my baby to sleep in a crib when he or she was older (6)
- Other (9)

Q35 At what age did your baby start sleeping overnight in a different room

▼ Less than 1 month (1) ... My baby is still sleeping in the same room (15)

Q36 If your baby was in bed with you, did you inform your pediatrician or family provider?

▼ Yes (1) ... No (2)

Q37 Did you experience postpartum depression?

- Yes (1)
- No (2)

Skip To: End of Survey If Did you experience postpartum depression? = No

Q38 Was your postpartum depression related to any of these, select all that apply

- Infant feeding (1)
- Sleep deprivation (2)
- Lack of adequate maternity leave (3)
- Lack of support at home (4)
- Lack of support at work (5)
- Other (6) ________________________________________________

Q39 Additional Comments?

________________________________________________________________

End of Block: YOUR BABY’S SLEEPING HABITS
