# Supplementary material for: Bedsharing among breastfeeding physicians: Results of a nationwide survey
Source: PLoS One. 2024 Aug 1;19(8):e0305625. doi: 10.1371/journal.pone.0305625 (PMC11293648; doi:10.1371/journal.pone.0305625)
Supplement: S2 Appendix — (DOCX) [file pone.0305625.s003.docx]

**S2 Appendix. Cox proportional hazards modeling**

We used Cox proportional hazards modeling in analyses. It is recommended that most people in a time-to-event analysis have the event observed, and in this study 73.1% had ceased breastfeeding by the time of the survey. Cox proportional hazards models are semi-parametric models that are widely used for time-to-event analyses because they do not require modeling the baseline hazard. The primary assumption in Cox proportional hazards model is that the hazard is proportional between the two comparison groups over time. The proportionality assumption was tested by the Schoenfeld residual test with global p = 0.061 on complete data, and Schoenfeld residual scaled plot. Both results showed the proportionality assumption was not violated. We also assessed the proportionality assumption for each covariate and found that it was not violated."

Table 1 Schoenfeld residual individual test result

| variable | Individual p value |
| --- | --- |
| bedsharing | 0.596 |
| race | 0.861 |
| ethnicity | 0.955 |
| Marital status | 0.763 |
| Trainee status | 0.619 |
| specialty | 0.090 |
| Birth year 2005 | 0.051 |
| Depression | 0.115 |

Figure 1 Scaled Schoenfeld residual plot


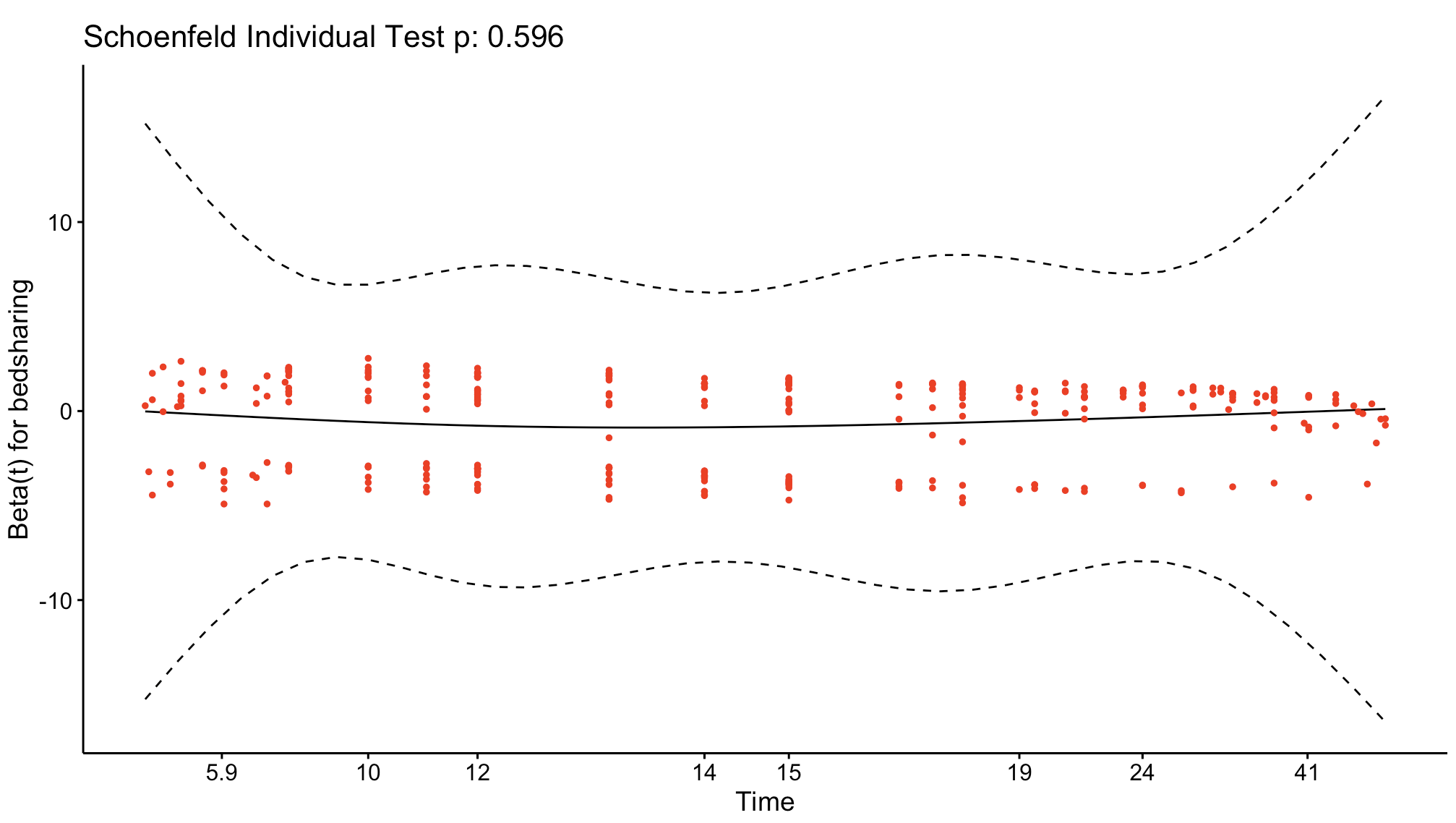


We adjusted for medical specialty, trainee status, race, Hispanic ethnicity, self-reported postpartum depression, and infant birth year before or after 2005 in the Cox proportional hazards regression. We did not have reason to suspect multicollinearity between these confounders and these were categorical or binary variables. Cox proportional hazards models are not ordinary least squares models and the confounders being categorical precludes a calculation of variation inflation factors as quantitative assessments of collinearity test.
