# Supplementary material for: Bedsharing among breastfeeding physicians: Results of a nationwide survey
Source: PLoS One. 2024 Aug 1;19(8):e0305625. doi: 10.1371/journal.pone.0305625 (PMC11293648; doi:10.1371/journal.pone.0305625)
Supplement: Attachment — Submitted filename: Bedsharing Reviewers Comments and Responses.docx [file pone.0305625.s005.docx]

January 15, 2024

Manuscript Number: PONE-D-23-16782

Manuscript Title: Bedsharing among breastfeeding physicians: Results of a nationwide survey

PLOS ONE

Dear Dr. Linglin Xie,

On behalf of my co-authors and myself, I would like to thank you and our reviewer for the prompt and thoughtful feedback on our manuscript. Attached, please find original comments and our responses. We are truly sorry for the delay in our response.

Reviewer #1: Dear Authors, you are come up with interesting title which creates a dilemma between d/t scholars. because some argue that bedsharing is important while others not. Additionally, your write up is smart however your paper have the following gaps:

1. Your summary doesn't contain introduction part which is not appropriate way

**Authors Response: Thank you very much. We have included an introduction in the abstract.**

2. your objective is not SMART which is not measurable. The verb you use not measurable.

**Authors Response: The objective in the abstract has been modified to be measurable. Thank you for this comment.**

3. You only use 3 kye words which is below standard.

**Authors Response: Thank you, we have increased the number of key words to 5 - breast feeding, lactation, sleeping habits, postpartum period, physicians.**

4. Methodology

a. Your methodology didn't go with your objective because your objective is to understand and to investigate the reason why bedsharing is high or low which is qualitative data. your study design didn't go with survival model. where is your follow up time??

**Authors Response: Studying the association between bedsharing and breastfeeding duration was one of the objectives. This outcome was a time-to-event variable with right censoring, so we elected to conduct time-to-event (survival) analysis. The follow-up time was the time from birth to the end of breastfeeding. Some participants were still breastfeeding when they took the survey, and these participants were censored. I can see why using a KM curve seems odd given that we did not follow a cohort of individuals, but rather surveyed responses at one time to determine "survival of breastfeeding" given bedsharing practice. The cross-sectional survey allowed retrospective data collection of time for a survival analysis.**

**The reasons for bedsharing were collected in the survey not as qualitative data. This was a secondary objective and was reported in table 5 to better understand the reasons for bedsharing or not bedsharing.**

b. Also, your sample size determination and procedure is not clear?? how you select 806 physician respondents or if you incorporate all physicians is there only 806 physicians in USA?? Think over??

**Authors Response: This is a cross sectional study design using a convenience sample of physicians who responded to the invitation to survey. We recruited as many participants as possible through 2 closed Facebook groups (Dr. MILK and Physician Moms Group) and Academy of Breastfeeding Medicine listserv. Recruitment was through social media platforms and by email listservs targeting breastfeeding physicians from October 2020 through July 2021.**

c. Why you include only single birth only?

**Authors Response: Multiples were excluded since that may confound the probability of breastfeeding continuation.**

5.result: you didn't make model fitness test, multicollinearity test and other important components were not performed

**We used Cox proportional hazards modeling in analyses. It is recommended that most people in a time-to-event analysis have the event observed, and in this study 73.1% had ceased breastfeeding by the time of the survey. Cox proportional hazards models are semi-parametric models that are widely used for time-to-event analyses because they do not require modeling the baseline hazard. The primary assumption in Cox proportional hazards model is that the hazard is proportional between the two comparison groups over time. The proportionality assumption was tested by the Schoenfeld residual test with global p = 0.061 on complete data, and Schoenfeld residual scaled plot. Both results showed the proportionality assumption was not violated. We also assessed the proportionality assumption for each covariate and found that it was not violated."**

Table 1 Schoenfeld residual individual test result

| **variable** | **Individual p value** |
| --- | --- |
| bedsharing | 0.596 |
| race | 0.861 |
| ethnicity | 0.955 |
| Marital status | 0.763 |
| Trainee status | 0.619 |
| specialty | 0.090 |
| Birth year 2005 | 0.051 |
| Depression | 0.115 |

Figure 1 Scaled Schoenfeld residual plot

**We adjusted for medical specialty, trainee status, race, Hispanic ethnicity, self-reported postpartum depression, and infant birth year before or after 2005 in the Cox proportional hazards regression. We did not have reason to suspect multicollinearity between these confounders and these were categorical or binary variables. Cox proportional hazards models are not ordinary least squares models and the confounders being categorical precludes a calculation of variation inflation factors as quantitative assessments of collinearity test.**

6 . what will be your interpretation of your outcome variable is it low or high requires justification??

**Authors Response: Thank you very much for this comment. We have expanded on this in the first paragraph of our discussion section.**

**Lines 249-253: “We found bedsharing was common among breastfeeding physicians consistent with our hypothesis, including physicians in specialties caring for women/mothers and/or infants. Also consistent with previous literature in the general population and our hypothesis, bedsharing is associated with longer duration of breastfeeding among physicians. There was a greater than 40% HR decrease for breastfeeding cessation.”**

**One would expect physicians to be among the most compliant groups to follow the AAP guidelines, given their type of work in the healthcare sector, and the fact that many are expected to counsel on this guideline as part of their job. This finding demonstrates that the recommendations are not consistent with the lived experiences of the physicians in our sample.**
